# Covalently Targeted Highly Conserved Tyr318 to Improve the Drug Resistance Profiles of HIV-1 NNRTIs: A Proof-of-Concept Study

**DOI:** 10.3390/ijms24021215

**Published:** 2023-01-07

**Authors:** Zhenzhen Zhou, Bairu Meng, Jiaqi An, Fabao Zhao, Yanying Sun, Dan Zeng, Wenna Wang, Shenghua Gao, Yu Xia, Caiyun Dun, Erik De Clercq, Christophe Pannecouque, Peng Zhan, Dongwei Kang, Xinyong Liu

**Affiliations:** 1 Key Laboratory of Chemical Biology (Ministry of Education), Department of Medicinal Chemistry, School of Pharmaceutical Sciences, Cheeloo College of Medicine, Shandong University, 44 West Culture Road, Jinan 250012, China; 2 Laboratory of Virology and Chemotherapy, Rega Institute for Medical Research, K.U. Leuven, Herestraat 49 Postbus 1043 (09.A097), B-3000 Leuven, Belgium; 3 China-Belgium Collaborative Research Center for Innovative Antiviral Drugs of Shandong Province, Shandong University, 44 West Culture Road, Jinan 250012, China

**Keywords:** HIV-1, NNRTIs, DAPYs, covalent inhibitors, drug resistance

## Abstract

This study presents proof of concept for designing a novel HIV-1 covalent inhibitor targeting the highly conserved Tyr318 in the HIV-1 non-nucleoside reverse transcriptase inhibitors binding pocket to improve the drug resistance profiles. The target inhibitor **ZA-2** with a fluorosulfate warhead in the structure was found to be a potent inhibitor (EC_50_ = 11–246 nM) against HIV-1 IIIB and a panel of NNRTIs-resistant strains, being far superior to those of NVP and EFV. Moreover, **ZA-2** was demonstrated with lower cytotoxicity (CC_50_ = 125 µM). In the reverse transcriptase inhibitory assay, **ZA-2** exhibited an IC_50_ value of 0.057 µM with the ELISA method, and the MALDI-TOF MS data demonstrated the covalent binding mode of **ZA-2** with the enzyme. Additionally, the molecular simulations have also demonstrated that compounds can form covalent binding to the Tyr318.

## 1. Introduction

Acquired immunodeficiency syndrome (AIDS) is an epidemic caused by HIV-1 infection, which seriously endangers human health. According to the latest data released by the World Health Organization (WHO), 38.4 million people worldwide are living with HIV-1, and 1.5 million people were newly infected with HIV-1 in 2021 [1]. The highly active antiretroviral therapy (HAART) is a combination of non-nucleoside reverse transcriptase inhibitors (NNRTIs), nucleoside reverse transcriptase inhibitors (NRTIs) and protease inhibitors (PIs) or integrase inhibitors (INTIs), which has transformed AIDS from a highly fatal disease to a chronic and manageable disease [2,3,4]. However, the long-term clinical use has produced a large number of HIV-1 mutant strains, which greatly reduced the therapeutic efficacy of the marked anti-HIV-1 drugs. HIV-1 NNRTIs are an important part of HAART due to their advantages of high efficiency and low toxicity. However, the most common NNRTIs-resistant strains, K103N and Y181C, cause 57% and 25% of the treatment failure of patients treated with NNRTIs [5]. Therefore, inhibitors with novel mechanisms of action are urgently needed to overcome the existing drug resistance.

Covalent inhibitors can covalently bind to the target proteins through special orientation and localization of covalent warheads, which have pharmacological advantages like smaller doses and prolonged duration of action [6]. More importantly, covalent binding can increase the affinity of small molecules to targets, thus improving resistance. Up to now, more than 50 covalent drugs have been approved by the Food and Drug Administration (FDA), and most of them are used as antitumor drugs. Recently, covalent inhibitors have been increasingly used in the antiviral field, such as severe acute respiratory syndrome coronavirus 2 (SARS-CoV-2), dengue virus, enterovirus, hepatitis C virus, human immunodeficiency virus, and influenza viruses. For example, the SARS-CoV-2 main protease covalent inhibitor, Nirmatrelvir (Pfizer), has been approved to treat COVID-19 [7]. As for HIV-1 NNRTIs, two classes of covalent RT inhibitors targeting Y181 and Y181C have been reported. However, a drawback of the former method is the difficulty in response to the Y181C, and the latter, though able to target Y181C, has the possibility of poor binding with WT RT and the other mutant strains. Furthermore, Y181 is the most easily mutated amino acid in NNIBP, and a variety of mutant strains have been reported, including Y181C, Y181V and Y181I. These reported RT covalent inhibitors showed excellent activity against wild-type HIV-1 strain, but greatly reduced activity against mutant strains [8,9,10]. Therefore, covalent inhibitors targeting conserved amino acids were proposed to improve the anti-drug resistance in this work.

**Figure 1:**
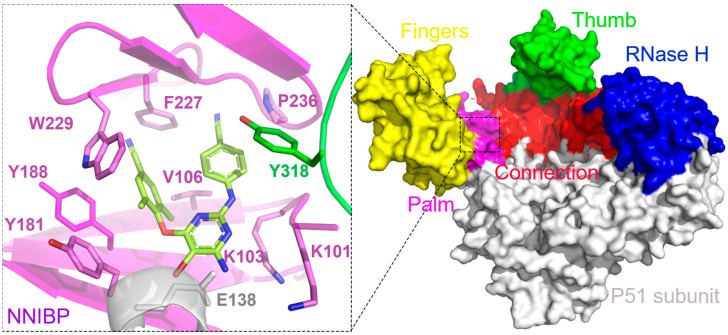
Cocrystal structure of etravirine/HIV-1 RT (PDB code: 3MEC) [11].

**Figure 2:**
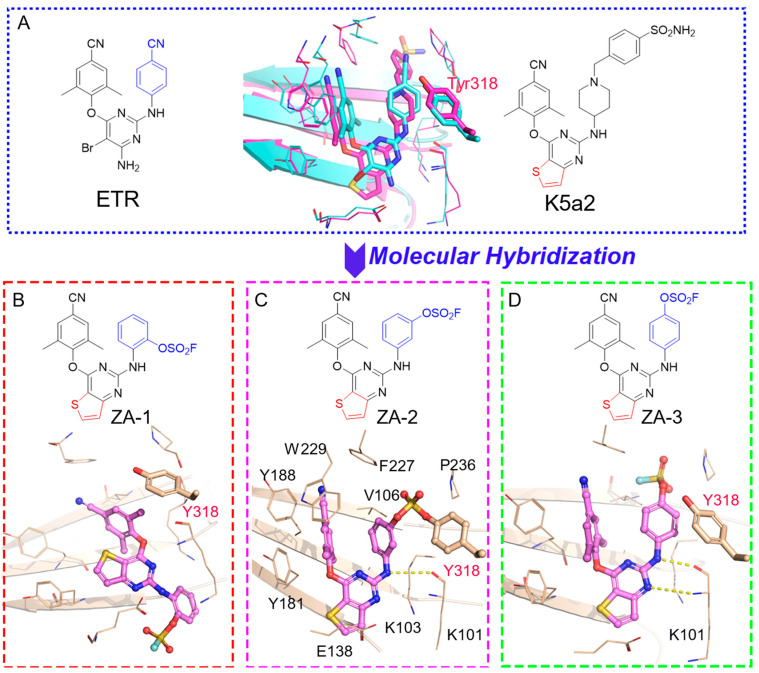
Design of the novel covalent inhibitors (**A**) and predicted binding modes of **ZA-1** (**B**), **ZA-2** (**C**), and **ZA-3** (**D**) with the HIV-1 WT RT (PDB code: 3MEC) [11]. All hydrogen bonds are shown as yellow dotted lines. Nonpolar hydrogen atoms are not shown for clarity.

NNRTIs binds to a hydrophobic pocket about 10 Å away from the polymerase active site, which is named the NNRTIs binding pocket (NNIBP) (Figure 1) [11]. NNIBP was composed by Leu100, Lys101, Lys103, Val106, Val179, Tyr181, Tyr188, Phe227, Trp229, Leu234, Pro236, Tyr318 of the p66 subunit and Glu138 of the p51 subunit. Among them, Tyr318 is a highly conserved amino acid, and no related mutant has been reported. Therefore, it can be used to design covalent inhibitors to improve the drug resistance profiles. Based on the superposition of etravirine (ETR)/HIV-1 RT and K-5a2/HIV-1 RT (Figure 2A), we abandoned the hERG-perturbing piperidine-linked benzenesulfonamide scaffold of K-5a2 and retained the benzene right wing of ETR, and oxysulfonyl fluoride covalent warhead was introduced to form a covalent binding with the highly conserved Tyr318. However, the central thiophene [3,2-*d*]pyrimidine ring has been identified as a privileged scaffold for improving the antiviral activity in our previous work, and it can more effectively occupy the NNIBP and form stronger interactions with RT [12,13,14,15,16,17,18,19]. Therefore, the dominant thiophene pyrimidine ring was introduced to replace the central pyrimidine ring of ETR based on the co-crystal structure superposition strategy (Figure 2B–D), and **ZA-1**, **ZA-2** and **ZA-3** with the fluorosulfate warhead at positions 2, 3 and 4 on the right-wing benzene group were designed.

Then, the three novel compounds were docked into the RT and predicted their binding mode. The results showed that the conformation of **ZA-1** in the NNBIP changes dramatically and cannot covalently bind to Tyr318 (Figure 2B), which may be due to the steric hindrance caused by the introduction of fluorosulfate warhead at the 2-position. While **ZA-3** maintained the classical horseshoe conformation and the fluorosulfate warhead arched into the tolerant region I, no covalent binding to Tyr318 was observed (Figure 2D). As for **ZA-2**, the result showed that the fluorosulfate warhead did covalently bind with Tyr318 (Figure 2C). In addition, **ZA-2** exhibited the following interactions with NNIBP: (i) The left wing extends into the tunnel formed by Tyr181, Tyr188, Phe227, and Trp229, and forms π-π interactions with these residues; (ii) the *N* atom of the pyrimidine forms the important hydrogen bond with the backbone of Lys101; (iii) the right wing projects into the tolerant region I, developing close van der Waals interactions with the lipophilic side chains of Lys103 and Val106. Based on the above molecular simulation analysis, **ZA-2** was synthesized and evaluated regarding its anti-HIV-1 activity against WT and mutant HIV-1 strains. Furthermore, the mechanism of **ZA-2** was also investigated.

## 2. Results and Discussion

### 2.1. Chemistry

**Scheme 1:**
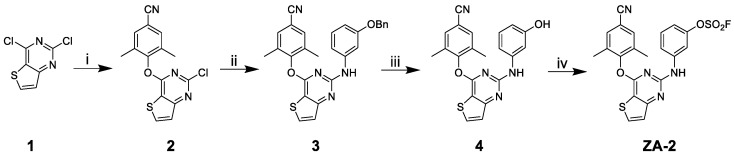
Synthesis of compound **ZA-2**. Reagents and conditions: (i) 3,5-dimethyl-4-hydroxybenzonitrile, K_2_CO_3_, DMF, r.t.; (ii) 3-benzyloxyaniline, BINAP, Pd_2_(dba)_3_, Cs_2_CO_3_, 1,4-dioxane, 90 °C; (iii) HBr, HOAc, 100 °C; (iv) fluorosulfuryl imidazolium triflate salt, Et_3_N, DCM, r.t.

The synthetic route of **ZA-2** is outlined in Scheme 1. The starting material 2,4-dichlorothieno[3,2-d]pyrimidine **1** was reacted with 3,5-dimethyl-4-hydroxybenzonitrile through nucleophilic substitution to obtain intermediate **2**, which was treated with 3-benzyloxyaniline to afford **3** via the Buchwald-Hartwig coupling reaction. Removing the Bn protecting group of **3** in the presence of HBr/HOAc gave the key intermediate **4**, which converted to **ZA-2** under the condition of fluorosulfuryl imidazolium triflate salt.

### 2.2. Anti-HIV-1 Activity

The anti-HIV-1 potency of **ZA-2** was evaluated in MT-4 cells infected with WT HIV-1 strain (IIIB) and NNRTIs-resistant strains, including single mutations L100I, K103N, Y181C, Y188L, E138K, and double mutation F227L + V106A. The first-generation drug nevirapine (NVP), efavirenz (EFV) and the second-generation drug ETR were selected as controls. The values of EC_50_ (anti-HIV-1 potency) and CC_50_ (cytotoxicity) of **ZA-2** were summarized in Table 1.

As shown in Table 1, **ZA-2** proved to a 11 nM inhibitor to HIV-1 IIIB, which was more potent than NVP (EC_50_ = 190 nM) and comparable to EFV (EC_50_ = 3.8 nM) and ETR (EC_50_ = 3.3 nM). As for K103N and F227L + V106A, **ZA-2** turned out to be potent inhibitor with EC_50_ values of 20 and 64 nM, being much superior to that of NVP and EFV. Against L100I and E138K, **ZA-2** exhibited EC_50_ values of 81 and 38 nM, which was more potent than that of NVP and comparable to that of EFV. In the case of Y181C and Y188L, **ZA-2** was demonstrated to have a decreased activity, yielding EC_50_ values of 159 and 246 nM, respectively. While **ZA-2** showed reduced potency against the selected NNRTIs-resistant strains, it exhibited much lower cytotoxicity (CC_50_ = 125 µM) and higher safety profiles.

### 2.3. Inhibition of HIV-1 RT

In order to validate the binding target, **ZA-2** was evaluated for its inhibitory ability to WT HIV-1 RT enzyme. As depicted in Table 2, the result demonstrated that **ZA-2** exhibited an IC_50_ value of 0.057 μM to HIV-1 RT, being comparable to that of NVP (IC_50_ = 0.150 μM) and ETR (IC_50_ = 0.010 μM). While the RT inhibitory activity of **ZA-2** was much inferior to that of EFV (IC_50_ = 0.004 μM), the result could demonstrate that the action target of **ZA-2** was HIV-1 RT.

### 2.4. Mass Spectrometry

**Figure 3:**
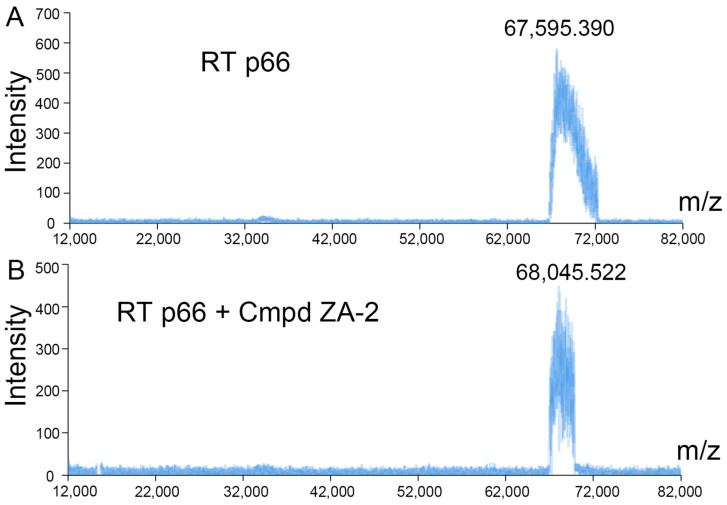
Representative spectra for MALDI-TOF MS analysis of RT covalent catalysis. (**A**) spectra for RT p66 protein; (**B**) spectra for RT p66 protein after incubation with **ZA-2**.

The WT HIV-1 RT p66 protein (10 μM+) was incubated alone or with **ZA-2** (1 μM) in 1‰ DMSO for 12 h at room temperature, and their molecular weights were determined by matrix-assisted laser desorption/ionization time of flight mass spectrometry (MALDI-TOF MS) to examine the potential covalent modification. As displayed in Figure 3, a peak with *m/z* value of 67595.390 Da was observed in the assay performed with RT p66 incubation alone, corresponding to RT p66 subunit (Figure 3A). Meanwhile, a peak with an *m/z* value of 68,045.522 Da was detected in the incubation of RT p66 and **ZA-2** with a mass shift of 450.132 (Figure 3B). The peak of 68,045.522 is the *m/z* of the covalent binding complex of RT p66 subunit (67,595.390) and **ZA-2** (470.05) with the disengagement of HF (20.008). The result demonstrated that the covalent binding of the compound **ZA-2** to HIV-1 RT, which confirmed **ZA-2**, was a covalent inhibitor acting on Tyr318.

### 2.5. Discussion

NNRTIs are a component of HAART regimens used to treat HIV-1. While six drugs have been approved by the FDA, the rapid emergence of drug resistance has limited their clinical use. Therefore, there is an urgent need to design inhibitors with novel mechanism of action to overcome the drug resistance. Covalent inhibitors design is a rapidly growing discipline in drug discovery, and a variety of covalent drugs approved in the field of anti-tumor. However, no covalent inhibitors have been approved for marketing in anti-HIV-1 drugs. Jorgensen W. L. et al. reported a class of Y181-target covalent NNRTIs, but these novel covalent inhibitors showed significantly decreased activity (EC_50_ = 160–280 nM) against HIV-1 IIIB [10]. We attributed the failure to the fact that Y181 is the amino acid with a high frequency of mutations in NNIBP and designing covalent inhibitors targeting the mutable amino does not improve the resistance of compounds. In fact, covalent inhibitors targeting conserved amino acids have been shown to be an effective strategy to improve resistance. The highly conserved amino acid Y318 was targeted to design novel covalent NNRTIs. As shown in Table 1, the target compound **ZA-2** exhibited potent activity against HIV-1 IIIB and some common NNRTIs-resistant strains. However, there are obvious differences in the efficacy between the reference drug EFV and **ZA-2** against the single mutant strains K103N, Y181C, E138K and double mutant strain F227L/V106A. To explain the difference in activity, the molecular docking simulation of **ZA-2** and EFV with these mutant strains were performed by Schrödinger software, and the structural visualizations were created by PyMOL 2.4.0. software.

**Figure 4:**
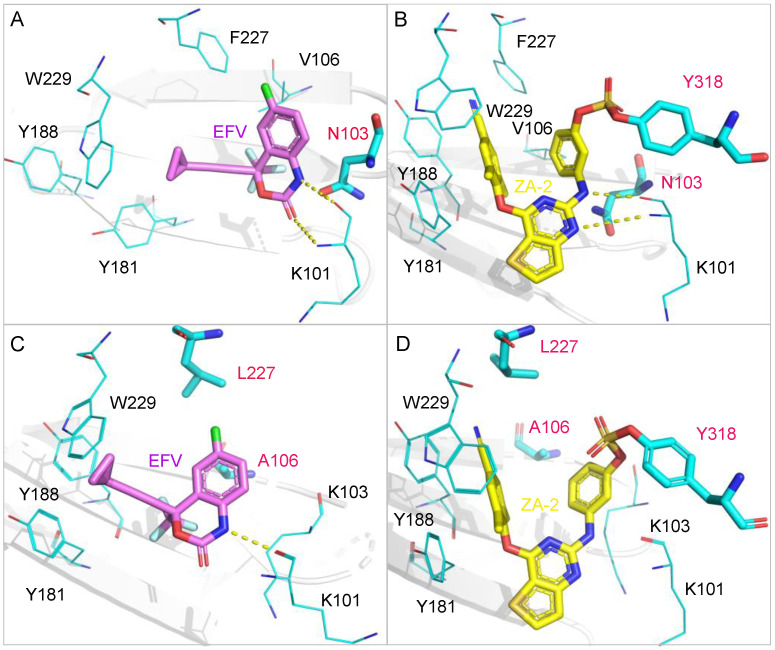
(**A**) The crystal structure of EFV and K103N HIV-1 RT and (**B**) predicted binding modes of **ZA-2** with K103N HIV-1 RT (PDB code: 1IKV), (**C**) EFV with F227L/V106A HIV-1 RT and (**D**) **ZA-2** with F227L/V106A HIV-1 RT (PDB code: 6DUF) [19,20]. All hydrogen bonds are shown as yellow dotted lines. Nonpolar hydrogen atoms are not shown for clarity.

**Figure 5:**
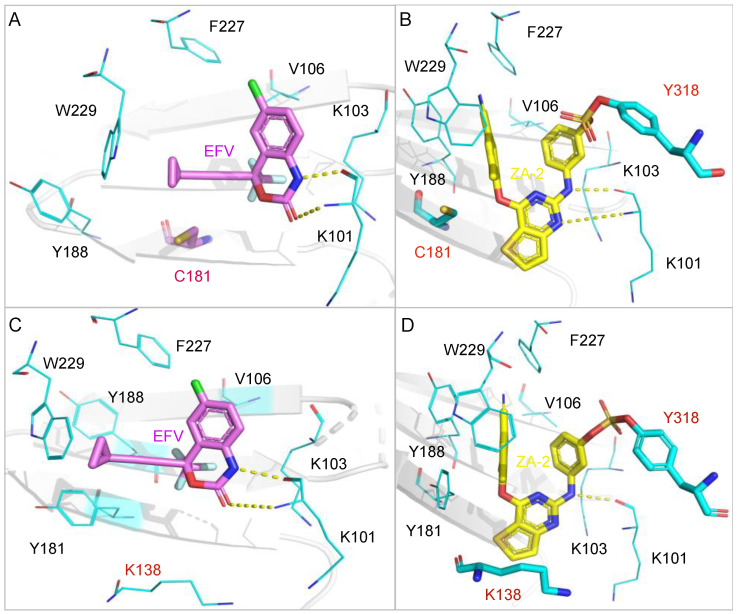
(**A**) The crystal structure of EFV and Y181C HIV-1 RT and (**B**) predicted binding modes of **ZA-2** with Y181C HIV-1 RT (PDB code: 1JKH), (**C**) EFV with E138K HIV-1 RT and (**D**) **ZA-2** with E138K HIV-1 RT (PDB code: 6C0P) [19,21]. All hydrogen bonds are shown as yellow dotted lines. Nonpolar hydrogen atoms are not shown for clarity.

Compared to EFV, the potency of **ZA-2** against the single mutant K103N and the double mutant F227L/V106A were increased by six-fold and four-fold, respectively. In the cocrystal structure of EFV/K103N RT, the replacement of K101 by N103 makes it far away from the EFV and thus eliminates the contact between K103 and the EFV, which resulted in the loss of van der Waals interactions and greatly reduced the potency of EFV against K103N (Figure 4A). In the binding model of EFV and F227L/V106A RT, the hydrogen bound between the carbonyl group of EFV and K101 disappeared due to the conformation change of the F227L/V106A mutant NNIBP, which may explain the decreased potency of EFV against F227L/V106A (Figure 4C). However, the flexibility of DAPYs skeleton enabled **ZA-2** to better adapt to the conformation changes of NNIBP caused by K103N or F227L/V106A. More importantly, the covalent binding of **ZA-2** and the conserved residue Y318 tightly anchored **ZA-2** in the NNIBP (Figure 4B,D), maintaining the conformation of **ZA-2**, which accounts for the good potency of **ZA-2** against K103N and F227L/V106A. As for the single mutant Y181C and E138K, the anti-HIV-1 potency of **ZA-2** showed 25.2-fold and 5.8-fold reduction than that of EFV, respectively. It is shown that the mutation removed the π-π stacking interactions in the left wing of **ZA-2** (Figure 5B), which was responsible for the reduced potency against Y181C. Figure 5D indicated that the E138K mutation would shove K101 away from **ZA-2**. The change in conformation in NNIBP would not only disrupt the hydrophobic interactions but also eliminate the hydrogen bound between pyrimidine and K101 due to the deviation of K101. However, in the cocrystal structure of EFV/ Y181C, the π-π stacking interactions between Y181 and EFV is so weak that the Y181C mutation has little effect on the inhibition potency of EFV (Figure 5A). In the case of the E138K mutant strain, the K138 residue would not shove K101 away from EFV due to the smaller skeleton of EFV (Figure 5C). Therefore, the two key hydrogen bonds between EFV and the K101 backbone were retained, thus maintaining the potent activity against the E138K mutant.

**Figure 6:**
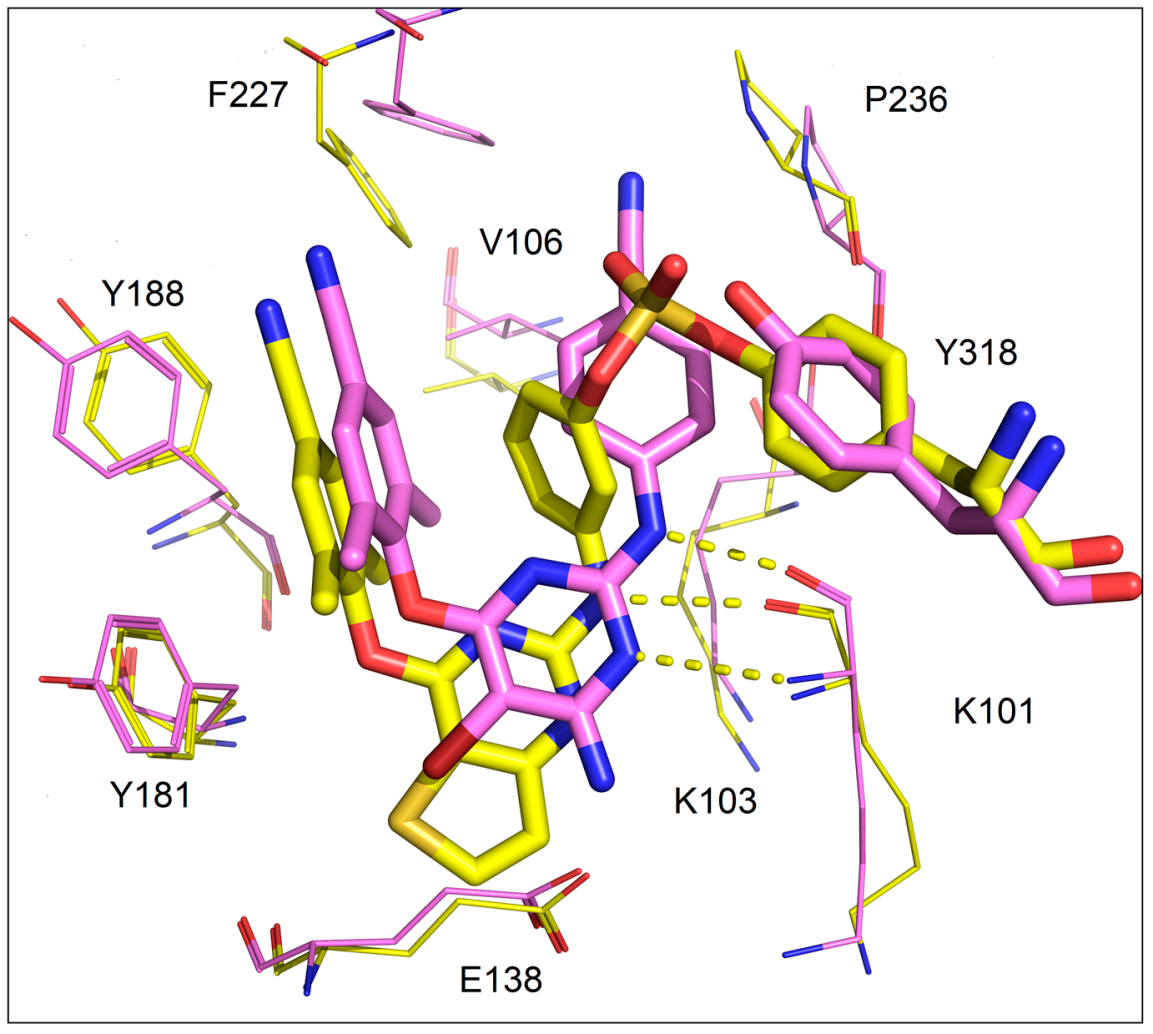
The superposition of ETR/HIV-1 RT co-crystal structure (purple) and the **ZA-2**/HIV-1 RT predicting binding model (yellow). All hydrogen bonds are shown as yellow dotted lines. Nonpolar hydrogen atoms are not shown for clarity.

Compared to ETR, the potency of **ZA-2** showed a two–nine-fold reduction against HIV-1 IIIB and the tested NNRTIs-resistant strains. The ETR/HIV-1 RT co-crystal structure and the **ZA-2**/HIV-1 RT predicting binding model was superimposed to explain the reason for the reduced potency. As shown in Figure 6, it can be found that **ZA-2** has an overall downward shift in NNIBP compared to that of ETR, which strengthens the hydrophobic interactions between the thiophene ring and Glu138, but disrupt the key hydrogen bound between pyrimidine and Lys101. Meanwhile, the localization effect of covalent binding of **ZA-2** to Tyr318 makes the right benzene ring of **ZA-2** rotate inward, and the **ZA-2** sub-pocket composed of Phe227, Val106, Pro236 and Tyr318 is displaced, thereby reducing the hydrophobic interaction between **ZA-2** and surrounding residues. While covalent binding can enhance the binding of the compound to HIV-1 RT, the lack of key interactions led to the reduced potency of **ZA-2**.

By analyzing the EFV co-crystal structures and **ZA-2** predicting binding models, we explained why their potency on different mutants vary greatly. Additionally, the ETR/HIV-1 RT cocrystal structure and the **ZA-2**/HIV-1 RT predicting binding model were superimposed to demonstrate the reduced potency of **ZA-2**. Moreover, the covalent binding of **ZA-2** to HIV-1 RT was proved by the HIV-1 RT inhibition experiment and MALDI-TOF MS experiment. Our further work will focus on the modification of phenylflurosulphate to form more extensive hydrophobic interactions between right wing and NNIBP and further improve drug resistance.

## 3. Methods and Materials

### 3.1. Chemistry

All reactions were routinely monitored by thin layer chromatography on Silica Gel GF254 (Merck) (Figure S1 in supplementary material). Flash column chromatography was performed on columns packed with Silica Gel (200–300 mesh, Haiyang Chemical Company, Qingdao, Shandong, CHN). Solvents (dichloromethane, ethyl acetate, *N*,*N*-dimethylformamide, 1,4-dioxane et al.) were obtained commercially and were purified and dried by standard methods. All melting points were determined on a micro melting point apparatus (RY-1G, TianGuang Optical Instruments, Tianjin, CHN). ^1^H NMR and ^13^C NMR spectra were recorded in DMSO-*d*_6_ on a Bruker AV-400 spectrometer with tetramethylsilane (TMS) as the internal standard (Figures S2 and S3 in supplementary material). Chemical shifts are reported in δ values (ppm) from TMS and coupling constants are given in hertz; signals are abbreviated as s (singlet), d (doublet), and m (multiplet). The mass spectra were measured in A G1313A Standard LC Autosampler (Agilent).

4-((2-Chlorothieno[3,2-*d*]pyrimidin-4-yl)oxy)-3,5-dimethylbenzonitrile (**2**)

Intermediate was prepared as previously reported [14].

4-((2-((3-(benzyloxy)phenyl)amino)thieno[3,2-*d*]pyrimidin-4-yl)oxy)-3,5-dimethylbenzonitrile (**3**)

A mixture of Pd_2_(dba)_3_ (0.072 g, 0.079 mmol) and BINAP (0.049 g, 0.079 mmol) in 10 mL of dry dioxane was stirred at room temperature for 10 min, and then 3-(benzyloxy)aniline (0.347 g, 1.74 mmol) and Cs_2_CO_3_ (0.773 g, 2.38 mmol) were added. Intermediate **2** (0.500 g, 1.58 mmol) was added to the mixture after an additional 10 min, and then the flask was evacuated and backfilled with nitrogen. The mixture was stirred at 90 °C for 8 h. Then solvent was evaporated under reduced pressure, and the obtained residue was dissolved in 20 mL of ethyl acetate (EA). The organic phase was washed with saturated sodium chloride (35 mL) and then dried over anhydrous Na_2_SO_4_, filtered, and purified by flash column chromatography to give the intermediate **3** as a white solid. Yield 47%, ESI-MS: *m/z* 479.10 [M + H]^+^; C_28_H_22_N_4_O_2_S (478.15).

4-((2-((3-hydroxyphenyl)amino)thieno[3,2-*d*]pyrimidin-4-yl)oxy)-3,5-dimethylbenzonitrile (**4**)

Compound **3** was added to 2 mL of 48% HBr and 2 mL of AcOH and heated at 100 °C for 1 h (monitored by TLC). After cooling to room temperature, 30 mL water was added, and the mixture was extracted with dichloromethane (3 × 10 mL). The organic phase was washed with saturated sodium chloride (10 mL), then dried over anhydrous Na_2_SO_4_ and purified by flash column chromatography to afford key intermediate **4** as a white powder, Yield 84%. ESI-MS: *m/z* 389.02 [M + H]^+^; C_21_H_16_N_4_O_2_S (388.10).

3-((4-(4-cyano-2,6-dimethylphenoxy)thieno[3,2-*d*]pyrimidin-2-yl)amino)phenyl sulfurofluoridate (**ZA-2**)

Fluorosulfuryl imidazolium triflate salt (0.101 g, 0.31 mmol) was added to a solution of **4** (0.100 g, 0.26 mmol) in dry DCM (10 mL), and stirred at room temperature overnight. Then 30 mL water was added, and the mixture was extracted with DCM (3 × 10 mL) and the organic phase was washed with saturated sodium chloride (10 mL). Dried over anhydrous Na_2_SO_4_ to give the corresponding crude product, which was purified by flash column chromatography and recrystallized from ethyl acetate (EA)/petroleum ether (PE) to give the target compound **ZA-2** as a white powder. Yield 49%. ^1^H NMR (600 MHz, DMSO-*d*_6_): δ 9.80 (s, 1H, NH), 8.38 (dd, *J* = 5.4, 1.3 Hz, 1H, C6-thienopyrimidine-H), 7.79 (s, 2H, Ph-H), 7.70 (d, *J* = 8.9 Hz, 2H, Ph-H), 7.45 (dd, *J* = 5.4, 1.1 Hz, 1H, C7-thienopyrimidine-H), 7.32 (d, *J* = 8.8 Hz, 2H, Ph-H), 2.16 (s, 6H, 2×CH_3_). ^13^C NMR (150 MHz, DMSO-*d*_6_): δ 165.28, 162.68, 157.71, 153.47, 143.83, 141.58, 138.11, 133.30, 133.24, 123.90, 121.26, 120.09, 118.86, 109.47, 108.07, 16.20. ESI-MS: *m/z* 471.28 [M + H]^+^; C_21_H_15_FN_4_O_4_S_2_ (470.05).

### 3.2. In Vitro Anti-HIV Assay

The 3-(4,5-dimethylthiazol-2-yl)-2,5-diphenyltetrazolium bromide (MTT) method was used to evaluate the anti-HIV-1 activity and cytotoxicity of **ZA-2**, NVP, EFV and ETR. The four stock (10 × final concentration) solution was added in 25 μL volume to two series of triplicate wells, respectively, to allow simultaneous evaluation of their effect on mock- and HIV-infected cells (obtained from Medical Research Council) [22,23]. Using a Biomek 3000 robot (Beckman instruments, Fullerton, CA, USA) made a serial 5-fold dilution (2 × final concentration) of the four stock solution in flat-bottomed 96-well microtiter trays, respectively, and taking untreated HIV- and mock-infected cell samples as controls. HIV-1 (IIIB) and mutant HIV-1 strains (50 μL) at 100–300 CCID50 (50% cell culture infectious doses) or culture medium was added to either the infected or mock-infected cell wells of the microtiter tray. Mock-infected cells were used to assess the effects of **ZA-2**, NVP, EFV and ETR on uninfected cells in order to evaluate the cytotoxicity of them. MT-4 cells were centrifuged for 5 min at 220 g, and then the supernatant was discarded. The MT-4 cells were resuspended at 6 × 105 cells/mL and 50 μL volumes were added to the microtiter tray wells (final concentration). 5 days post-infection, the viability of mock- and HIV-infected cells was evaluated spectrophotometrically by the MTT method. Absorbance was read in an eight-channel computer-controlled luminometer at wavelengths of 540 and 690 nm, respectively. All data were calculated using the median absorbance value of three wells. The 50% cytotoxic concentration (CC_50_) was defined as the concentration of the compounds that reduced the absorbance (OD540) of the mock-infected control sample by 50%. The concentration achieving 50% protection against the cytopathic effect of the virus in infected cells was defined as the 50% effective concentration (EC_50_).

### 3.3. HIV-1 RT Inhibition Assay

The RT assay kit produced by Roche was used to perform the HIV-1 RT inhibition assay [24]. The procedure of experiments was conducted as the kit protocol. The complex of reconstituted template, the HIV-1 RT enzyme and viral nucleotides [digoxigenin (DIG)- dUTP, biotin-dUTP, and dTTP] was incubated for 1 h at 37 °C with the incubation buffer in the presence or absence of **ZA-2**. The reaction mixture was then transferred to streptavidin-coated microtiter plates (MTP) and incubated at 37 °C for 1 h. After that, the unbound dNTPs were detached by the washing buffer, and the anti-DIG-PODs were added. After an additional 1 h incubation at 37 °C, unbound anti-DIG-PODs were removed and a solution of peroxide substrate (ABST) was added to the MTPs. The reaction mixture was incubated at 25 °C until the green color was sufficiently developed for detection. The absorbance of the sample was measured at OD450 using a microtiter plate ELISA reader. The percentage of inhibitory activity of RT inhibitors was calculated by the following formula: Inhibition (%) = (OD value with RT but without inhibitors-OD value with RT and inhibitors)/OD value with RT and inhibitors-OD value without RT and inhibitors.

### 3.4. Molecular Simulation Studies

The target protein HIV-1 RT (PDB: 3MEC, 1IKV, 6DUF, 1JKH and 6C0P) was obtained from RCSB Protein Date Bank [11]. The water molecules were removed, while a RT structure in the asymmetric unit was extracted and optimized using the Protein Preparation Wizard module in Schrödinger software. A grid box of 20 Å × 20 Å × 20 Å centered on the active site was generated as the docking area using the Receptor Grid Generation module. Subsequently, the 3D structure of **ZA-2** was prepared in the Ligprep module, while the ionized states of **ZA-2** (pH = 7.4 ± 1.0) were generated using the Epik method. The aforementioned RT structure and the conformations of **ZA-2** were allowed to dock in the Glide module. Next, the best binding modes were chosen according to the glide docking scores and used for subsequent covalent docking. The parameters were set as follows: the Tyr318 residue and **ZA-2** were assigned as the reaction partners and a Michael reaction was assigned as the reaction type. Structural visualizations were created in PyMOL 2.4.0.

### 3.5. Mass Spectrometry

Matrix-assisted laser desorption/ionization-time of flight (MALDI-TOF) mass spectrometry was used to determine the molecular weight of complexes of compound **ZA-2** with WT HIV-1 RT p66 protein. The enzyme at a final concentration of 10 µM was incubated with the inhibitor (1 µM) in 50 mM Tris-HCl pH 8.0, containing 50 mM NaCl, 5 mM MgCl_2_ and 3.3% DMSO at 37 °C during 60 min. Then, inhibitor and salts were removed by centrifugal ultrafiltration (10,000 molecular weight cut-off membranes, Microcon-10, Amicon, Merck Millipore). The sample (15 µL) was diluted 20-fold with ultrapure distilled water and centrifuged at 14,000× *g* for 6 min. The process was repeated several times until the absorbance of the filtrate was below 0.001, in the range of 220–300 nm. The desalted protein was dried, resuspended in 30 µL of aqueous 2% trifluoroacetic acid (TFA) solution. Samples were diluted at 1:1 ratio (*v*/*v*) with matrix solution (50% saturated sinapinic acid in 70% aqueous acetonitrile and 0.1% trifluoroacetic acid). A 1.0 µL aliquot of this mixture was manually deposited onto a 386-well OptiTOFTM Plate (ABSciex, Foster City, CA, USA) and allowed to dry at room temperature.

Molecular weights were determined by using an ABi 4800 MALDI TOF/TOF mass spectrometer (SCIEX, Foster City, CA, USA) in positive ion linear mode (the ion acceleration voltage was 25 kV for MS acquisition). The detection mass range was set between 12,000 and 82,000 *m/z*.

## 4. Conclusions

To sum up, this proof-of-concept study presents the discovery of the first HIV-1 RT covalent inhibitor targeting the highly conserved Tyr318. Based on the co-crystal structure superposition of ETR/RT and K-5a2/RT, the fluorosulfate warhead was introduced to the right wing of the privileged hybrid skeleton, with the aim to form covalent binding with Tyr318 and improve the drug resistance profiles. The anti-HIV-1 results demonstrated that the target inhibitor **ZA-2** showed potent activity against HIV-1 IIIB, L100I, K103N, E138K and F227 + V106A, with EC_50_ values of 11 nM, 81 nM, 20 nM, 38 nM, and 64 nM, respectively. Additionally, **ZA-2** exhibited much lower cytotoxicity (CC_50_ = 125 µM) than that of NVP, EFV and ETR. Then, the reverse transcriptase inhibition assay and mass spectrometry were also conducted to confirm the covalent binding of **ZA-2** with RT.

## Figures and Tables

**Table 1:** Anti-HIV-1 activity and cytotoxicity of **ZA-2**.

Compd.	EC_50_ (nM) ^a^							CC_50_ (µM) ^b^
	IIIB	L100I	K103N	Y181C	Y188L	E138K	F227L + V106A	
**ZA-2**	11 ± 5.1	81 ± 17	20 ± 8.1	159 ± 11	246 ± 38	38 ± 5.4	64 ± 1.8	125 ± 3.8
**NVP**	190 ± 76	2233 ± 982	6039 ± 2657	7405 ± 1783	>6125	218 ± 123	>2141	>15
**EFV**	3.8 ± 0.7	53 ± 27	119 ± 24	6.3 ± 2.0	247 ± 55.8	6.5 ± 1.7	255 ± 103	>6.3
**ETR**	3.3 ± 0.38	12.4 ± 5.1	4.3 ± 1.0	18.1 ± 3.8	25 ± 7.6	18 ± 7.2	25 ± 7.2	>4.6

^a^ EC_50_: concentration of compound required to achieve 50% protection of MT-4 cell cultures against HIV-1-induced cytotoxicity, as determined by the MTT method. ^b^ CC_50_: concentration required to reduce the viability of mock-infected cell cultures by 50%, as determined by the MTT method.

**Table 2:** Inhibitory activity to WT HIV-1 RT of **ZA-2**.

Compd	ZA-2	NVP	EFV	ETR
**IC_50_ (μM) ^a^**	0.057 ± 0.006	0.150 ± 0.060	0.004 ± 0.001	0.010 ± 0.000

^a^ IC_50_: inhibitory concentration of test compound required to inhibit biotin deoxyuridine triphosphate (biotindUTP) incorporation into WT HIV-1 RT by 50%.

## Data Availability

The data presented in this study are available on request from the corresponding author.

## Supplementary Materials

The following supporting information can be downloaded at: https://www.mdpi.com/article/10.3390/ijms24021215/s1.
