# Supplementary material for: Covalently Targeted Highly Conserved Tyr318 to Improve the Drug Resistance Profiles of HIV-1 NNRTIs: A Proof-of-Concept Study
Source: Int J Mol Sci. 2023 Jan 7;24(2):1215. doi: 10.3390/ijms24021215 (PMC9865928; doi:10.3390/ijms24021215)
Supplement: Supplementary file 1 [file ijms-24-01215-s001.zip › ijms-2041808-supplementary.pdf]

Support Information

Covalent Inhibition of Wild-Type HIV-1 Reverse Transcriptase with a Fluorosulfate Warhead Targeting the Residue Tyr318

Zhenzhen Zhou[1], Bairu Meng[1], Jiaqi An[1], Fabao Zhao[1], Yanying Sun[1], Dan Zeng[1], Wen'na Wang[1], Shenghua Gao[1], Yu Xia[1], Caiyun Dun[1], Erik De Clercq[2], Christophe Pannecouque[2], Peng Zhan[1,3], Dongwei Kang[1,3,*] and Xinyong Liu[1,3,*]

1 Key Laboratory of Chemical Biology (Ministry of Education), Department of Medicinal Chemistry, School of Pharmaceutical Sciences, Cheeloo College of Medicine, Shandong University, 44 West Culture Road, 250012 Jinan, Shandong, PR China

2 Rega Institute for Medical Research, Laboratory of Virology and Chemotherapy, K.U. Leuven, Herestraat 49 Postbus 1043 (09.A097), B-3000 Leuven, Belgium

3 China-Belgium Collaborative Research Center for Innovative Antiviral Drugs of Shandong Province, Shandong University, 44 West Culture Road, 250012 Jinan, Shandong, PR China

* Correspondence: kangdongwei@126.com (D.K.); xinyongl@sdu.edu.cn (X.L.)

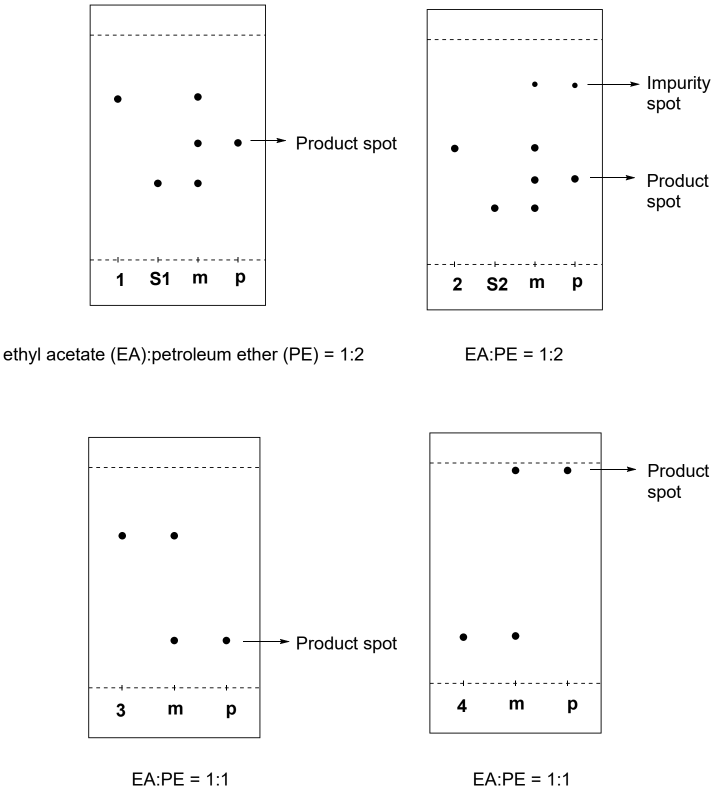

Figure S1: The TLC detection of intermediates **2**, **3**, **4** and compound **ZA-2**. m: mixing point of materials and peoducts. p: products.

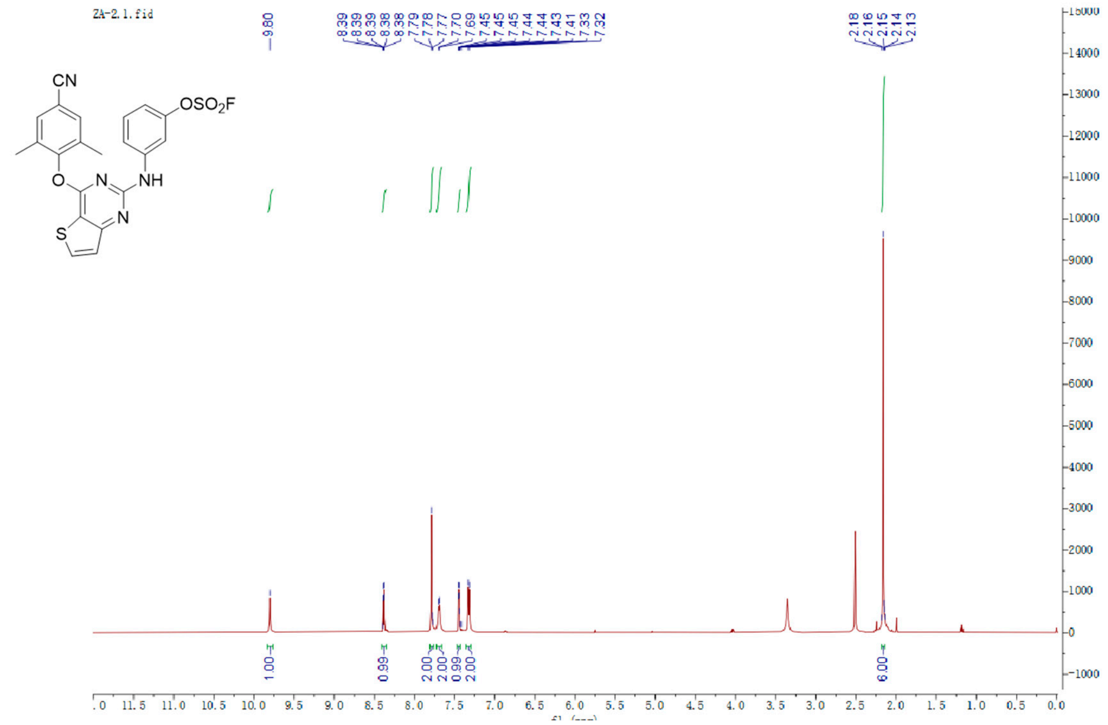

Figure S2: The ^{1}H NMR spectrum of **ZA-2**.

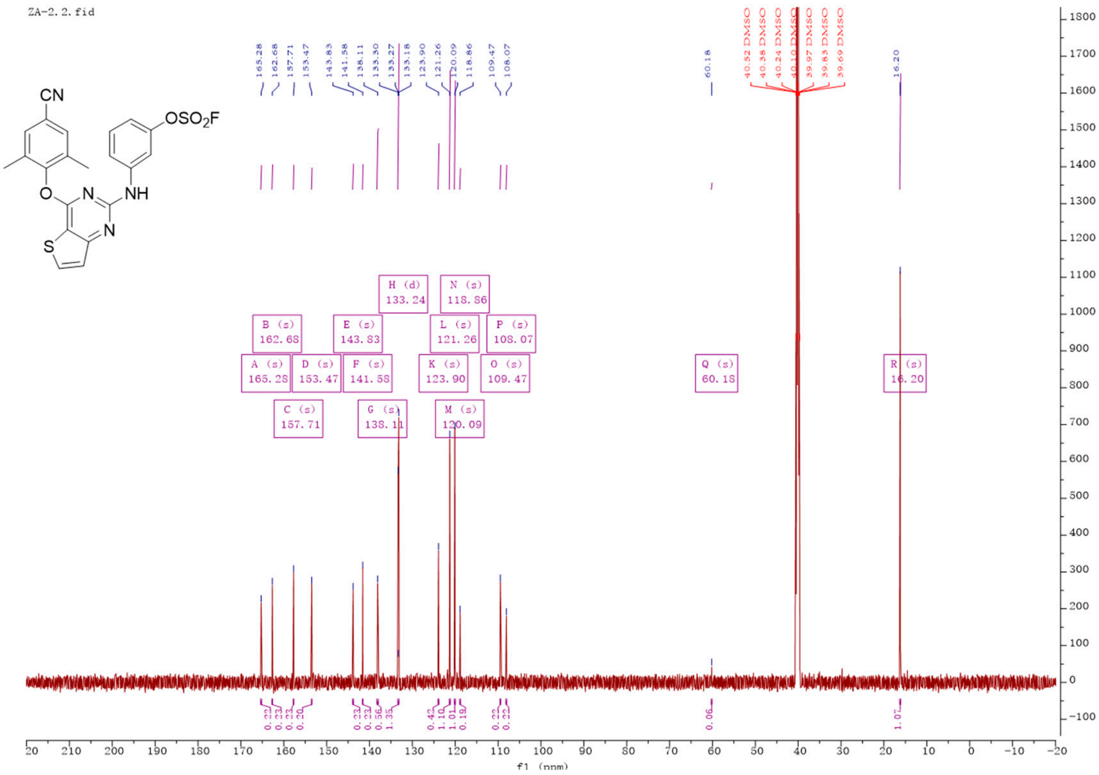

Figure S3: The ^{13}C NMR spectrum of **ZA-2**.
